# Laser Acupuncture Research: China, Austria, and Other Countries—Update 2018

**DOI:** 10.3390/medicines5030092

**Published:** 2018-08-20

**Authors:** Gerhard Litscher

**Affiliations:** Research Unit for Complementary and Integrative Laser Medicine, Research Unit of Biomedical Engineering in Anesthesia and Intensive Care Medicine, and TCM Research Center Graz, Medical University of Graz, Auenbruggerplatz 39, EG19, 8036 Graz, Austria; gerhard.litscher@medunigraz.at; Tel.: +43-316-385-83907; Fax: +43-316-385-595-83907

**Keywords:** laser acupuncture, research, countries, China, Austria, USA

## Abstract

This editorial contains an overview of the current status of published articles (pubmed) on the subject of laser acupuncture research. Ordered by country, a rough analysis is carried out.

The number of studies on laser acupuncture listed in SCI and PubMed databases is steadily increasing. Altogether, in PubMed, the most important medical database (www.pubmed.gov), there are over 900 publications on this topic, as of August 2018. Although the practice of laser acupuncture in China still seems to be in its infancy, China occupies the first place for published research in the international scientific ranking. A total of 225 scientific papers on the subject of laser acupuncture with author participation from China were published. If one looks at the details, it is worth noting that 44 articles were produced with the participation of the Traditional Chinese Medicine (TCM) Research Center of Graz (chairman: G. Litscher). In fact, Austria plays a leading role in laser acupuncture, together with China (see Figure 1). In addition, it has to be mentioned that there are about 100 scientific papers published in the Russian language.

Remarkable is the fact that the Austrian researchers have published more articles in this study area than the USA and German researchers together. If one goes a bit deeper in this analysis, one recognizes that of the 98 published articles from Austria, a very high percentage originates from researchers of the Graz TCM Research Center (*n* = 91). Therefore, it is not an exaggeration to state that Graz has developed into a hotspot for laser acupuncture research and that, together with the representatives of ISLA (International Society for Medical Laser Applications, Germany), it will set the course for future priorities.

One important issue will be the development of automatically individualized dose adjustment in laser acupuncture, which is not currently realizable by any commercially available device. The author of this editorial has repeatedly pointed out in national and international lectures that the ideas for producing such devices are available, but, so far, no company appears to be willing to implement them and build individual device components. It is to be hoped that this implementation will be carried out by industrial partners who recognize the potential of these products, which is supported not only by eminence-based but also by evidence-based research.

At the end of this year (2018), the 4th ISLA Asian conference on Medical Laser Therapy and Regenerative Medicine in Bangkok, Thailand, will take place (see www.isla-laser.org and www.litscher.info). Laser Acupuncture [1,2,3] is gaining a special boost in Thailand; therefore, research on this subject will be given special emphasis at this conference.

We, the two presidents of ISLA (Dr. Michael H. Weber and Prof. Gerhard Litscher), would be pleased to welcome you personally in Bangkok (29 November–1 December 2018; translation into Chinese will be available). An exciting event awaits you.

## Figures and Tables

**Figure 1:**
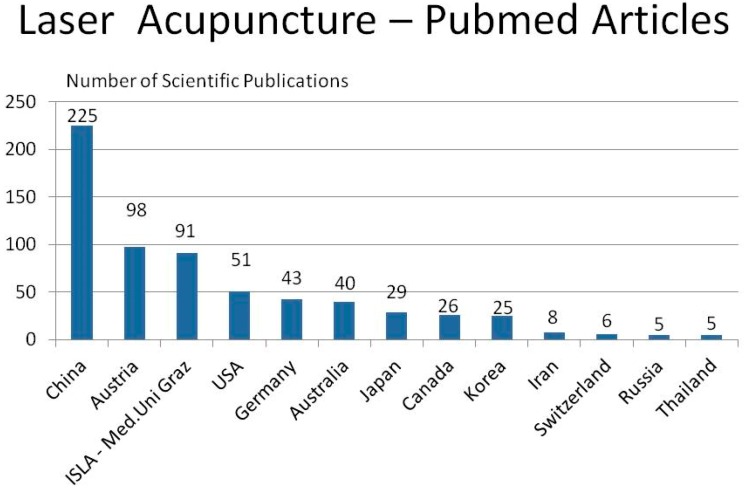
Ranking of countries according to the number of published scientific articles (the majority in the English language) on laser acupuncture.
